# A test for reporting bias in trial networks: simulation and case studies

**DOI:** 10.1186/1471-2288-14-112

**Published:** 2014-09-27

**Authors:** Ludovic Trinquart, John PA Ioannidis, Gilles Chatellier, Philippe Ravaud

**Affiliations:** INSERM U1153, Hôpital Hôtel-Dieu, 1 place du Parvis Notre-Dame, 75004 Paris, France; Centre Cochrane Français, Paris, France; Department of Epidemiology, Columbia University Mailman School of Public Health, New York, NY USA; Stanford Prevention Research Center, Department of Medicine and Department of Health Research and Policy, Stanford University School of Medicine, Stanford, CA USA; Department of Statistics, Stanford University School of Humanities and Sciences, Stanford, CA USA; Meta-Research Innovation Center at Stanford (METRICS), Stanford University, Stanford, CA USA; Université Paris Descartes - Sorbonne Paris Cité, Paris, France; INSERM CIE 4, Paris, France; Assistance Publique-Hôpitaux de Paris, Hôpital Européen Georges Pompidou, Unité de Recherche Clinique, Paris, France; Assistance Publique-Hôpitaux de Paris, Hôpital Hôtel-Dieu, Centre d’Epidémiologie Clinique, Paris, France

**Keywords:** Publication bias, Selective outcome reporting, Test of bias, Randomized controlled trials, Comparative effectiveness research

## Abstract

**Background:**

Networks of trials assessing several treatment options available for the same condition are increasingly considered. Randomized trial evidence may be missing because of reporting bias. We propose a test for reporting bias in trial networks.

**Methods:**

We test whether there is an excess of trials with statistically significant results across a network of trials. The observed number of trials with nominally statistically significant p-values across the network is compared with the expected number. The performance of the test (type I error rate and power) was assessed using simulation studies under different scenarios of selective reporting bias. Examples are provided for networks of antidepressant and antipsychotic trials, where reporting biases have been previously demonstrated by comparing published to Food and Drug Administration (FDA) data.

**Results:**

In simulations, the test maintained the type I error rate and was moderately powerful after adjustment for type I error rate, except when the between-trial variance was substantial. In all, a positive test result increased moderately or markedly the probability of reporting bias being present, while a negative test result was not very informative. In the two examples, the test gave a signal for an excess of statistically significant results in the network of published data but not in the network of FDA data.

**Conclusion:**

The test could be useful to document an excess of significant findings in trial networks, providing a signal for potential publication bias or other selective analysis and outcome reporting biases.

**Electronic supplementary material:**

The online version of this article (doi:10.1186/1471-2288-14-112) contains supplementary material, which is available to authorized users.

## Background

Reporting bias proceeds from the tendency of researchers, pharmaceutical companies and journals to publish trial results based on the direction, magnitude and statistical significance of the results [1, 2]. It is still a major concern for meta-analysts because, if statistically significant “positive” results are more likely available, a meta-analysis based on these results will be biased [3]. In particular, selective analysis and outcome reporting biases are increasingly being considered as more common and thus a potentially greater threat than publication bias [4].

Numerous statistical tests have been introduced to detect the presence of reporting bias in conventional meta-analyses [5–11]. Most formally assess the extent of asymmetry in funnel plots as a sign of small-study effects, the tendency for smaller trials to show larger treatment effect estimates, whatever the reasons [12, 13]. Instead of testing whether smaller trials yielded different treatment effect estimates than did larger trials, Ioannidis and Trikalinos proposed testing for an excess number of trials with statistically significant results in a meta-analysis [14]. Unlike for other tests, the performance of this test has not been evaluated by simulation studies under different scenarios and thus the Cochrane Collaboration has not yet recommended its use in conventional meta-analyses [15]. The test is underpowered in cases of only a few trials with significant results in a meta-analysis, which is a common scenario in single meta-analyses of randomized trials. However, the major advantage of the test is that it can be used to examine large bodies of evidence from many meta-analyses, and identify biases that pertain to such larger bodies of evidence. This feature sets it apart from other proposed tests of selective reporting bias. Thus the test has been used in evaluating bias across bodies of evidence including dozens to hundreds of meta-analyses in diverse fields such as genetics, brain volume abnormalities, and biomarkers [16–20].

The classic situation of many meta-analyses of randomized trials with the same setting, disease and outcome is network meta-analysis [21–23]. The pairwise meta-analyses that are combined in a network meta-analysis address different treatment comparisons, but the trials are considered to be similar (“coherent”) enough for analysis under the same network. Reporting bias can also be a major, and probably neglected, concern in network of trials and their meta-analyses [24, 25]. Networks of trials and their meta-analyses are increasingly being used [26]. Although reporting bias may have an important impact on treatment efficacy estimates, many published network meta-analyses fail to address formally or discuss the possibility of publication bias and related small-study bias [27, 28]. Moreover, there is a need to develop reliable tests for reporting bias across an entire network of trials. A test providing statistical signals, provided it is used soundly, would complement and enhance network meta-analyses as a systematic effort to summarize evidence stemming from a network of trials. We propose to apply the Ioannidis-Trikalinos test in networks of trials. Because the test was originally introduced and further used to explore research domains, it can be readily used for trial networks [17–19, 29, 30].

## Methods

### Test of bias in a conventional meta-analysis

We consider a meta-analysis of *n* trials. We test whether *O*, the observed number of trials with statistically significant results at a specified *α* level, differs from *E*, the expected number of trials with statistically significant results. We set *α*=0.05, the traditional threshold for inference in randomized trials.

To estimate the expected number *E*, we assume a true effect size *θ*. The expected probability that trial *i* will find a statistically significant “positive” result equals its power 1-*β*_*i*_ to detect *θ* at the specified *α* level. The expected number *E* equals the sum of the power estimates across all trials selected in the meta-analysis, .

The true effect size *θ* for any meta-analysis is not known. Thus, we must make assumptions about a plausible effect. Such plausible effects could be: the fixed-effect summary, the random-effects summary or the treatment effect estimate of the largest trial in the meta-analysis, respectively. One may also consider not just a point estimate but also a prior distribution for the plausible effect. However, prior work has shown that results are very similar to using a point estimate [14], which is what we use here.

We test whether the observed number *O* is greater than the expected number *E* at a statistical level *α*^′^ using a binomial probability test. We set *α*^′^=0.10, as is typical for selective reporting bias tests. Consequently, we would reject the null hypothesis in favor of excess significant findings if , where *p*=*E*/*n* is the average probability that a specific trial will find a “positive” result and  is the binomial distribution function. Because the test is based on observed and expected numbers of “positive” results, it can be used for binary, continuous or time-to-event outcome data.

For binary outcome data, let us assume that we observe *x*_*Ei*_ and *x*_*Ci*_ events in *n*_*Ei*_ and *n*_*Ci*_ patients in the experimental and control groups of trial *i*, respectively. We estimate the true proportion of event in the control group by  and the true proportion of events in the experimental group by , with *θ* the true odds-ratio between the experimental and control groups. Then, 1-*β*_*i*_ is estimated as the power of the two-sided Fisher’s exact test to detect the difference between  and  in *n*_*Ei*_ and *n*_*Ci*_ patients at the specified *α* level. *O* is the number of trials with significant p-value at a significance level *α* with a two-sided Fisher’s exact test based on *x*_*Ei*_, *x*_*Ci*_, *n*_*Ei*_, *n*_*Ci*_.

For continuous outcome data, let us assume that we observe the means and standard deviations *m*_*Ei*_, *s*_*Ei*_ and *m*_*Ci*_, *s*_*Ci*_ from *n*_*Ei*_ and *n*_*Ci*_ patients in the experimental and control groups, respectively, of trial *i*. The true mean in the control group is estimated by  and the true mean in the experimental group is estimated by , with *g* the true standardized mean difference between the experimental and control groups and  the within-groups standard deviation, pooled across groups. Then, 1-*β*_*i*_ is estimated as the power of the two-sided t-test to detect a difference between *μ*_*Ei*_ and *μ*_*Ci*_ in *n*_*Ei*_ and *n*_*Ci*_ patients at the specified *α* level. *O* is the number of trials with significant p-value at a significance level of *α* with a two-sided t-test based on *m*_*Ei*_, *s*_*Ei*_, *n*_*Ei*_ and *m*_*Ci*_, *s*_*Ci*_, *n*_*Ci*_.

### Test of bias in a network of trials

We consider that the network of trials can be described as *J* meta-analyses of *n*_*j*_ trials each. We estimate the expected number *E*_*j*_ of trials with statistically significant results for each meta-analysis across the network by assuming a true effect size *θ*_*j*_ for each meta-analysis. The expected number *E* is estimated as . Estimation of the true effect size for each meta-analysis is based on a plausible effect size, as above. The observed number  is the total number of trials with significant p-values across the network. We test whether the observed number *O* is greater than the expected number *E* using a one-tail binomial probability test. Let . We would reject the null hypothesis in favor of excess significant findings if , where *P*=*E*/*N* is the average probability that a specific trial across the network will find a “positive” result.

Detection of excess significant findings does not mean that all pairwise meta-analyses included in the network have been equally affected by the same bias. Even if the bias is exchangeable across all pairwise meta-analyses in the network, we may observe that some pairwise meta-analyses are affected more than others, and some are not affected at all. For instance, for 3 meta-analyses with 10 trials per meta-analysis, and because of reporting bias, 10% of the evidence disappears in a “file drawer”, it is within the range of chance that these 3 file-drawer trials may come one from each meta-analysis, or all 3 may come from the same meta-analysis. However, detection of excess significance suggests that selective reporting bias may have affected the results of this whole body of evidence and thus inferences should be cautioned in this regard.

### Simulation studies

We assessed the type I error rate and power of the test using Monte Carlo simulation studies. These simulations were based on binary outcome data. The protocol for simulation studies is described in detail in the Additional file 1.

For a given meta-analysis (i.e., a given pair of experimental and control treatments), we set the number of trials *n*, the true average treatment effect as *θ* and the between-trial variance *τ*^2^ and we generated the number of events and non-events in the experimental and control groups for each trial under a random-effects model. We used *n* as (6, 10, 30); *θ* as (0.5, 0.75, 1.0); *τ*^2^ as (0.02, 0.08, 0.25).

To simulate reporting bias affecting trials in a given meta-analysis, we considered a selection model that links the probability of trial selection to both trial size and intensity of treatment effect [9, 31, 32]. A correlation parameter *ρ* defines the extent of reporting bias. When we induced reporting bias, we simulated data until *n* trials had been selected. As sensitivity analysis, we also consider a model that selects trials depending on the p-value for treatment effect associated with the trial [5–7].

We simulated a network of trials as *J* meta-analyses of *n*_*j*_=*n* trials each. For each meta-analysis *j*, we set the true average treatment effect *θ*_*j*_. We considered 4 distinct scenarios by setting known relative effects from  with *ψ* equal to *ψ*_1_= log(0.75) or *ψ*_2_= log(0.95) and *ν* equal to *ν*_1_=0.02 or *ν*_2_=0.08. The sets of realizations are reported in Table 1. We further assumed homogeneity of between-trial variation within the *J* meta-analyses, i.e., . We took *J* as (6, 10), *n* as (3, 6), *θ*_*j*_ as in Table 1, and *τ*^2^ as (0.02, 0.08, 0.25). These values were based on the characteristics of a large sample of Cochrane meta-analyses [33, 34]. For a given meta-analysis, data were then generated as described.

**Table 1:** **True average treatment effects** ***θ***_***j***_ **used to generate networks of trials**

Treatment	Dispersion of	No. of	True average treatment effects
effect***ψ***	treatment effect***ν***	meta-analyses***J***	***θ***_***j***_,***j***=1,⋯,***J***
*ψ*1= log(0.75)	*ν*_1_=0.02	6	0.793, 0.889, 0.741, 0.954, 0.569, 0.684
*ψ*1= log(0.75)	*ν*_2_=0.08	6	0.808, 0.725, 0.876, 0.698, 0.699, 0.395
*ψ*2= log(0.95)	*ν*_1_=0.02	6	0.796, 1.172, 1.000, 1.171, 1.099, 0.883
*ψ*2= log(0.95)	*ν*_2_=0.08	6	0.491, 1.214, 0.936, 0.977, 1.451, 0.754
*ψ*1= log(0.75)	*ν*_1_=0.02	10	0.658, 0.852, 0.696, 0.889, 0.741, 0.722, 0.645, 0.683, 0.816, 0.796
*ψ*1= log(0.75)	*ν*_2_=0.08	10	0.978, 0.432, 1.149, 0.706, 0.432, 0.751, 0.679, 0.653, 0.624, 0.568
*ψ*2= log(0.95)	*ν*_1_=0.02	10	1.081, 1.089, 0.767, 0.973, 0.781, 0.763, 1.266, 0.816, 1.066, 0.992
*ψ*2= log(0.95)	*ν*_2_=0.08	10	0.845, 0.637, 1.030, 0.799, 0.541, 0.851, 1.063, 1.307, 0.674, 0.732

We set the relative effects *θ*_*j*_,*j*=1,…,*J* from .

Reporting bias was induced for each of the *J* meta-analyses constituting the network as described, under the assumption that the propensity for bias would be similar across the *J* meta-analyses. When selecting trials according to trial size and treatment effect magnitude, we drew each *ρ*_*j*_ from an uniform distribution over the support [ *ρ*_*min*_;*ρ*_*max*_]. We considered  or  to reflect similar moderate and severe bias, respectively, across the *J* meta-analyses.

For each scenario, we generated 10,000 datasets. We assessed the empirical type I error rate and power for scenarios without and with reporting bias, respectively. We took into account the possibly differing type I error rates of the tests and we estimated powers adjusted for type I error rate [35]. Moreover, we estimated the likelihood ratio of a positive test result (ie, the likelihood that a significant test result is found in networks with bias as opposed to networks without bias) and the likelihood ratio of a negative test result (ie, likelihood that a non-significant test result is found in networks with bias as opposed to networks without bias) [36]. Analyses involved use of R v2.12.2 (R Development Core Team, Vienna, Austria).

In the cases of a conventional meta-analysis, we also assessed for comparison purposes the performance of the test introduced by Rücker et al. based on a weighted regression of the arcsine transformation of observed risks with explicit modeling of between-trial heterogeneity [9].

### Application of the test with 2 trial networks

**Figure 1:**
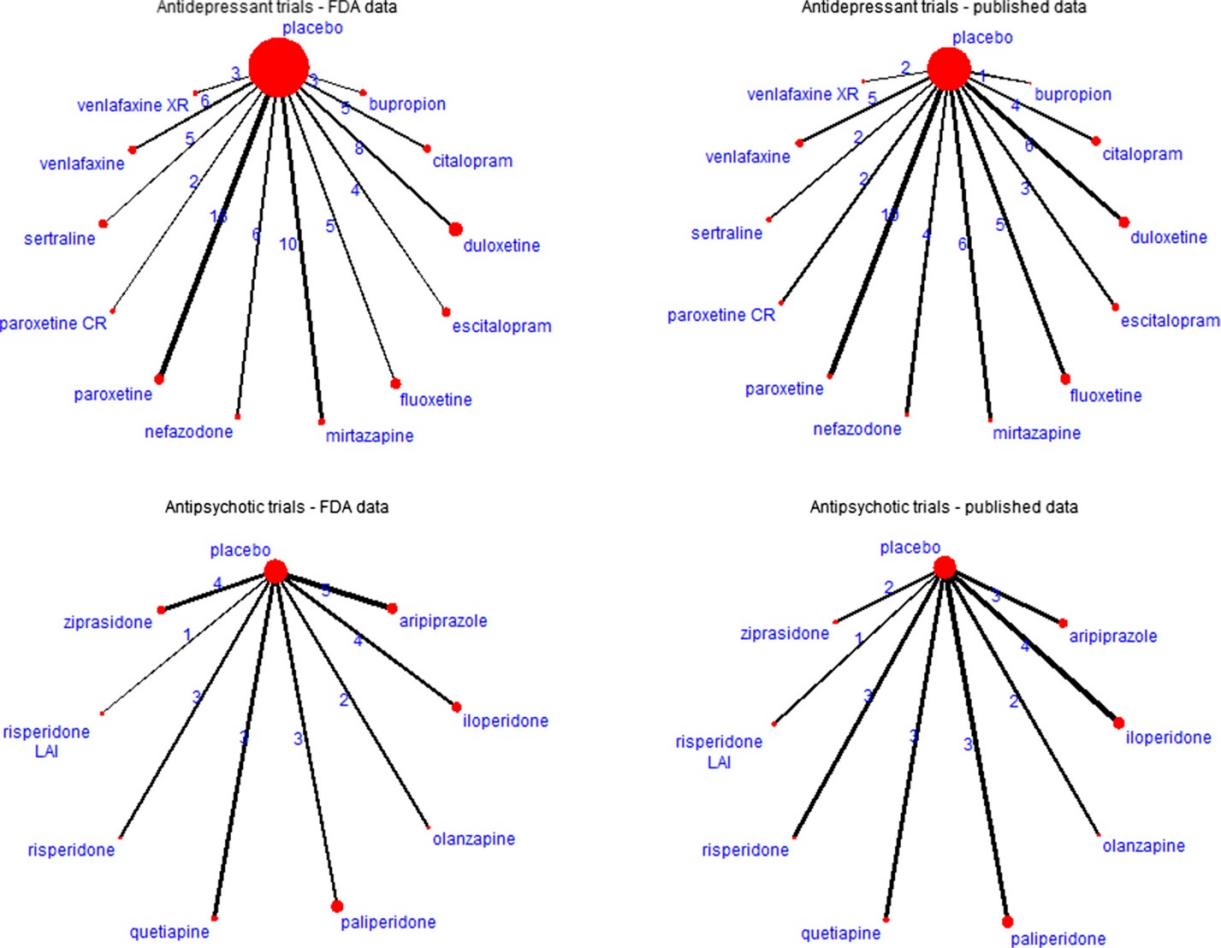
**Networks of antidepressant and antipsychotic trials.**

We provide two illustrations of the test for networks of antidepressant and antipsychotic trials where strong and weak selective reporting biases have been convincingly demonstrated based on a comparison of FDA data versus published data (Figure 1).

#### Networks of antidepressant trials

For the antidepressant trials, we used 2 star-shaped networks created from US Food and Drug Administration (FDA) reviews of antidepressant trials and their matching publications [37]. Turner et al. originally identified all randomized placebo-controlled trials of 12 antidepressant drugs approved by the FDA and then all publications matching these trials. The authors identified 74 trials registered with the FDA but only 51 trials had published results. They showed that the 23 entire trials remained with unpublished results because of the negative nature of the results. Moreover, in some journal articles, specific analyses were reported selectively and effect sizes differed from that in FDA reviews. The outcome was the change from baseline to follow-up in depression severity score. The measure of effect was a standardized mean difference. To illustrate the test, we used the effect sizes reported by Turner et al. (table C in the Supplementary Appendix from [37]). Because the results of 2 paroxetine CR (controlled release) trials with published results were combined, it made a total of 73 trials registered to the FDA and 50 trials with published results for our assessment.

#### Networks of antipsychotic trials

Turner et al. also used FDA data to assess whether the apparent efficacy of second-generation antipsychotics had been influenced by reporting bias [38]. The authors identified the phase 2/3 randomized placebo-controlled trials of 8 antipsychotic drugs approved by the FDA and then all publications matching these trials. The authors identified 24 trials registered with the FDA, among which 20 had published results. The outcome was the change from baseline to follow-up in schizophrenia symptom ratings. The measure of effect was a standardized mean difference. We used the effect sizes reported by Turner et al. (Tables S2 and S3 in Supplementary Appendix from [38]). Because the results of one risperidone trial was published as two separate trials, it made a total of 25 trials registered to the FDA and 21 trials with published results for our assessment.

## Results

### Simulation studies in a conventional meta-analysis

Complete results in the cases of a conventional meta-analysis are reported in the Additional file 2. Briefly, the proposed test showed type I error inflation when heterogeneity was substantial (between-trial variance of 0.25) and/or with a large number of trials per meta-analysis (30 trials). On the contrary, the arcsine test introduced by Rücker et al. maintained a better type I error rate across all scenarios. With low number of trials per meta-analysis, all tests had low power. When the true odds ratio was 0.5, the heterogeneity low or moderate and the number of trials large, the proposed test, with the true effect size estimated as the fixed-effect or random-effects summary, had acceptable power. In all other cases, because of the inflation of the type I error rate, the power adjusted for type I error rate of the proposed test was clearly insufficient. When selection of trials was modeled by trial size and intensity of treatment, the most powerful test was the arcsine test.

### Simulation studies in a network of trials

#### Type I error rate

**Figure 2:**
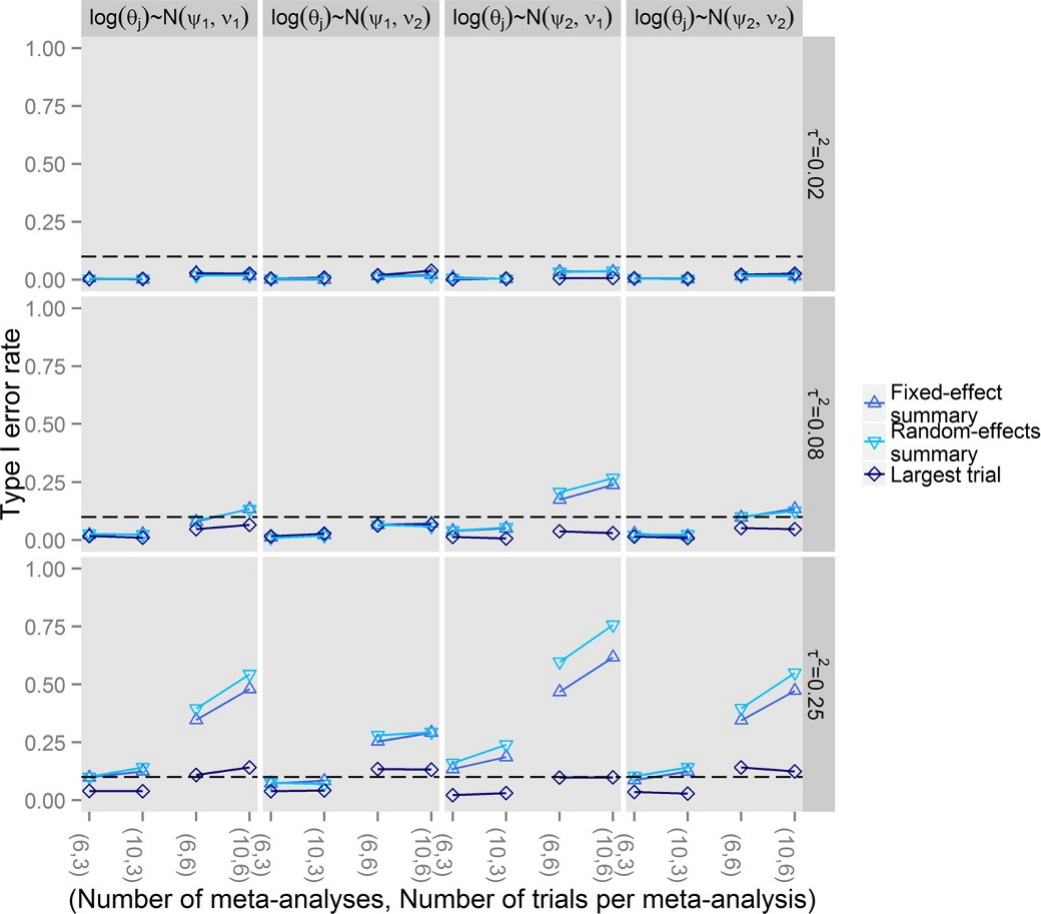
**Type I error rate of the extended tests for reporting bias in a network of trials.**

Results for scenarios without reporting bias are presented in Figure 2. The empirical type I error rate increased with increasing heterogeneity and increasing numbers of meta-analyses and trials per meta-analysis. Whatever the vector of true average treatment effects, the proposed test was too conservative with low between-trial variance within a meta-analysis (*τ*^2^=0.02). Conversely, error inflation was substantial with substantial between-trial variance within a meta-analysis (*τ*^2^=0.25) except when the true effect size was estimated as the treatment effect estimate of the largest trial in the meta-analysis with the empirical type I error rate being in good agreement with the pre-specified significance level of 0.10.

#### Power

**Figure 3:**
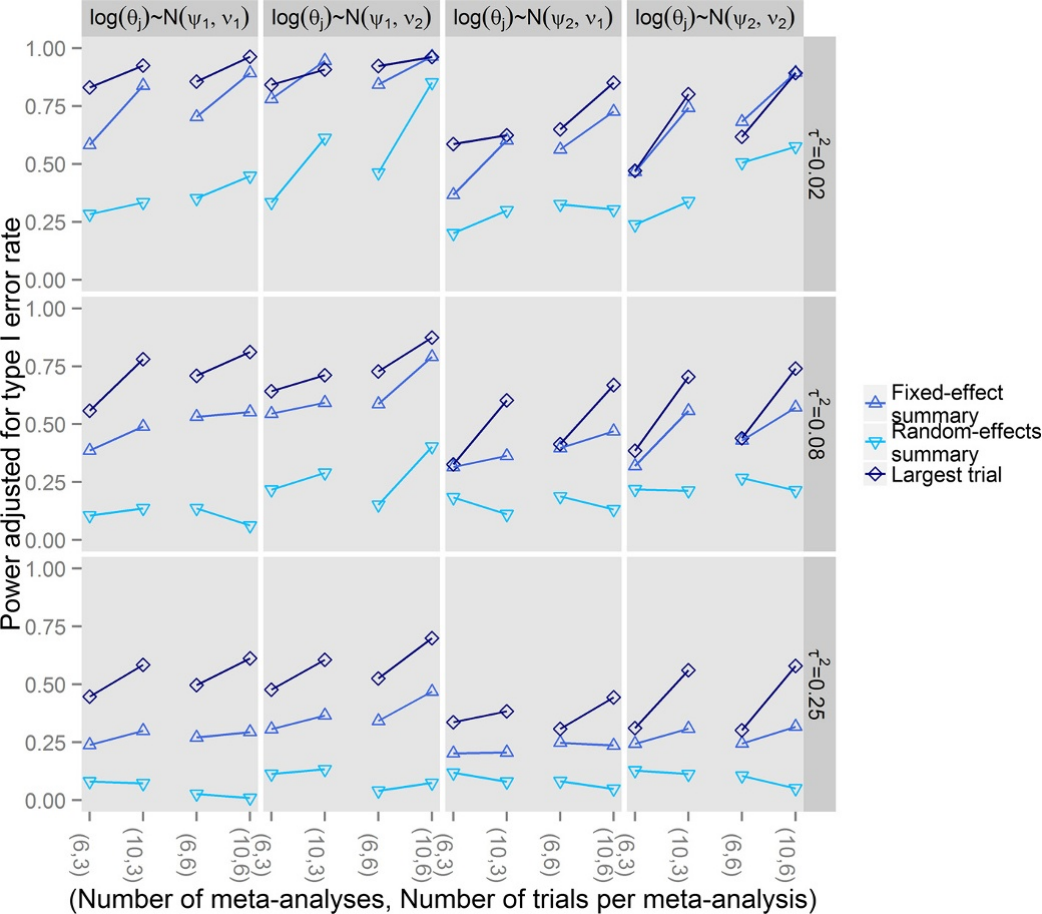
**Adjusted power of the extended tests for reporting bias in a network of trials.**

Results for scenarios with reporting bias are presented in Figure 3 (each ). Across all scenarios, visual inspection of the trellis graphs showed that the power adjusted for type I error rate increased with increasing number of meta-analyses and trials per meta-analysis and with decreased between-trial variance within a meta-analysis. With 6 meta-analyses and 3 trials per meta-analysis, all tests had low to moderate power. When selection of trials was modeled by trial size and treatment effect magnitude, the power adjusted for type I error rate increased when the true average odds ratios moved away from 1. The proposed test with the true effect size estimated as the treatment effect estimate of the largest trial in the meta-analysis showed greater adjusted power than with the fixed-effect summary and the latter showed greater adjusted power than with the random-effects summary. Results for other extents of bias were similar (Additional file 3). When selection of trials was modeled by p-value, the adjusted power was still the smallest when the true effect size was estimated as the random-effects summary, while fixed effects and largest trial effect sizes had relatively similar performance (Additional file 3).

We also ran simulations with another set of vectors of true average treatment effects *θ*_*j*_. Results were similar (not shown).

#### Likelihood ratio

**Figure 4:**
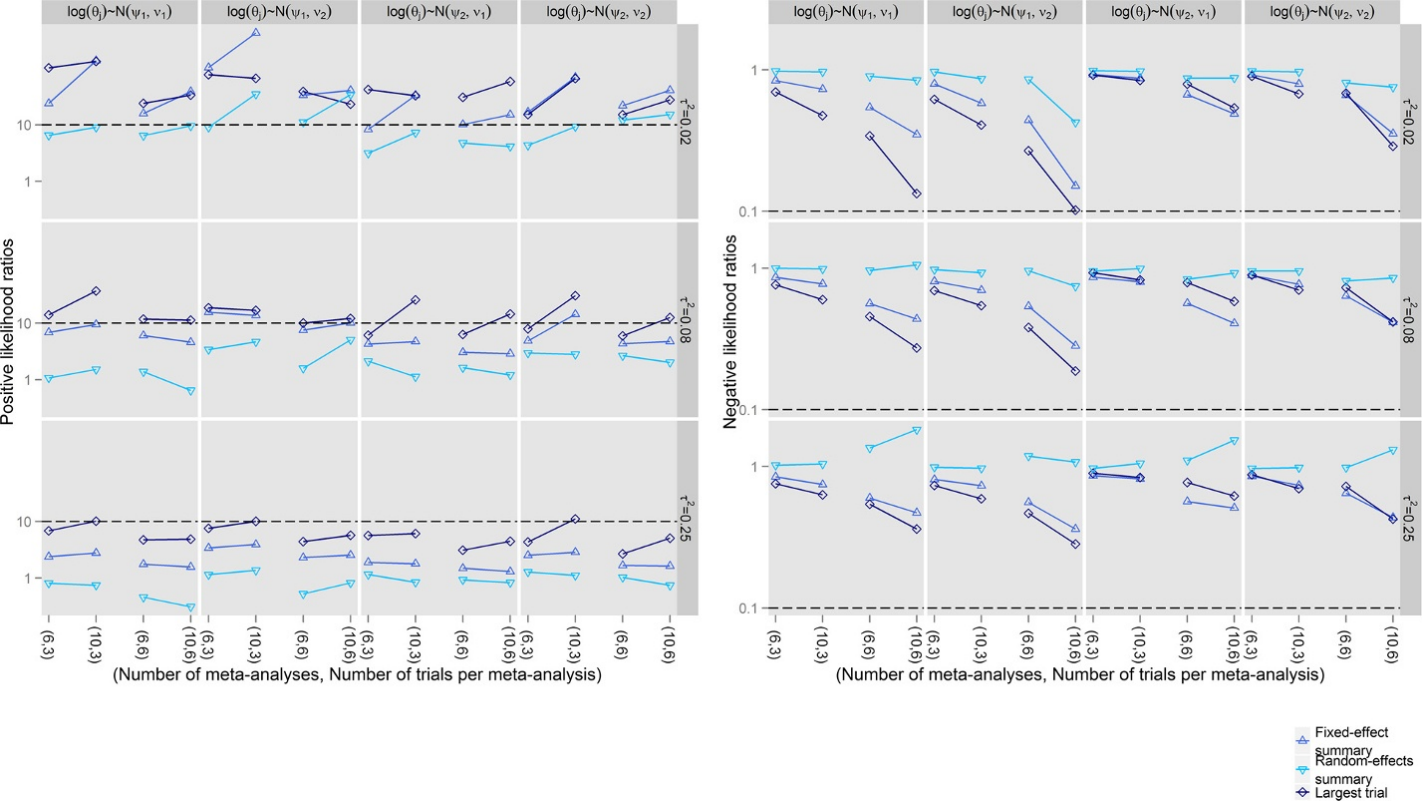
**Likelihood ratios of the extended tests for reporting bias in network of trials.**

Likelihood ratios of a positive test result indicated that the proposed test had modest (with substantial heterogeneity) or high (with little or no heterogeneity) effect on increasing the likelihood of bias. Likelihood ratios of a negative test result indicated that the proposed test had a weak to moderate effect in decreasing the likelihood of bias (Figure 4). The proposed test with the true effect size estimated as the estimate of the largest trial yielded the best likelihood ratios. The performance of the test was poor with the true effect estimated as the random-effects summary.

### Application of the test with 2 trial networks

#### Networks of antidepressant trials

When we considered the fixed-effect summaries as the plausible effect sizes, the observed number of trials with significant results across the network of published data was larger than the expected number (Table 2). This excess was statistically significant at the pre-specified level of 0.10 (binomial probability test *p*=0.05) which may indicate bias in trials with published results. However, across the network of FDA data, we found no signal of an excess of statistically significant results (*p*=0.24). Results were similar when using random-effects summaries as plausible effect sizes. However, when using the estimates of the largest trials as plausible effect sizes, there was still an even stronger signal for an excess of statistically significant results in the published data and there was a signal even in the network of FDA data. The latter signal is not necessarily a false-positive: even though the FDA data are more complete and unbiased than the published data, it is possible that some bias may have existed even in the FDA data [39].

**Table 2:** **Application of the test with networks of antidepressant and antipsychotic trials**

Antidepressant trials						
	Published data			FDA data		
	(N =51 trials)			(N =74 trials)		
Plausible effects	***O***	***E***	***p***	***O***	***E***	***p***
Fixed-effect summary	41	35.3	0.05	38	34.5	0.24
Random-effects summary	41	35.6	0.06	38	34.6	0.25
Largest trial	41	31.3	0.002	38	29.0	0.02
**Antipsychotic trials**						
	**Published data**			**FDA data**		
	**(N =20 trials)**			**(N =24 trials)**		
**Plausible effects**	***O***	***E***	***p***	***O***	***E***	***p***
Fixed-effect summary	19	18.1	0.43	20	19.6	0.53
Random-effects summary	19	18.4	0.50	20	34.6	0.56
Largest trial	19	16.1	0.10	20	18.6	0.36

*O* and *E* are the observed and expected total numbers of trials with statistically significant results across the network; *p* is the p-value associated with a one-tail binomial probability test to asssess if *O* is greater than the *E*.

#### Networks of antipsychotic trials

When we considered the fixed-effect summaries as the plausible effect sizes, we found no evidence of an excess of statistically significant results across the network of published data and the network of FDA data. Results were similar when using random-effects summaries as plausible effect sizes. However, when using the estimates of the largest trials as plausible effect sizes, there was a signal for an excess of statistically significant results in the network of published data but not in the network of FDA data.

## Discussion

In this paper, we proposed a test for reporting bias in networks of trials. The test is based on the observed and expected numbers of trials with statistically significant results across the network. In simulation studies, we found that the type I error rate of the proposed test was in agreement with the nominal type I error level and that the proposed test had overall moderate power after adjustment for type I error rate, except when the between-trial variance was substantial in which case the empirical type I error rate was considerably inflated and the empirical power was low. In all, a positive test result increases modestly or even markedly the probability of reporting bias being present, while a negative test result is not very informative.

The proposed test fits well with the widespread notion that a treatment effect estimate has to pass a cut-off of statistical significance, resulting in an aversion to null results or, conversely, in significance chasing [40, 41]. Although the test does not make any assumption about small-study effects, publication bias and related types of small-study effects may explain the excess significance, with bias being more pronounced in smaller trials. For instance, in an empirical assessment of animal studies of neurological diseases, the strongest excesses of significance were found in meta-analyses where small-study effects were found [42]. Finally, an excess of significant results could reflect suppression of results of entire trials but also selective analysis and outcome reporting practices, or even fabrication of positive results (although this latter scenario is likely more rare for clinical trials) [43]. In fact, reporting bias encompasses several mechanisms that may act solely or simultaneously, but all may be motivated by statistical significance.

In a network of trials, conventional tests for asymmetry could be applied to each meta-analysis constituting the network. If reporting bias is detected in any pairwise comparison, meta-analysts have a signal that they should interpret the synthesis results with caution. However, the number of trials addressing each pairwise comparison may often be limited (<10 trials for each pairwise comparison), which would prevent this approach from documenting or excluding reporting bias appropriately [30–32]. Moreover, testing one-at-a-time the multiple meta-analyses comprising the network may result in some false-positive asymmetry test result simply by chance. Here, the proposed test involves a single evaluation of the entire network, thus avoiding this multiplicity. Moreover, it borrows strength from trials across the network under the assumption that biases are exchangeable, that is biases, if present, operate in a similar way in trials across the network. As discussed elsewhere, if we have no information to distinguish different reporting bias mechanisms across the network, bias exchangeability is plausible and it still allows that eventually some meta-analyses will be affected by bias more than others within the range afforded by chance [44].

Decisions about conclusiveness and dissemination of research findings are commonly based on statistical significance (only) and this practice is likely to affect any network of evidence. We acknowledge that the exchangeability assumption may not be tenable in contexts in which reporting biases may affect the network in a systematically unbalanced way. For instance, only some pairwise comparisons could be affected and not others. However, the proposed test could still be useful in such networks if there are sufficient numbers of trials across the affected pairwise comparisons.

To estimate the expected number of trials with statistically significant results, estimates of the unknown true effect sizes for each meta-analysis in the network are required. In this regard, the true effect sizes are approximated by pooled estimates using just the very trials that are suspected to be affected by a meta-bias. However, we note that, in the presence of reporting bias, the fixed-effect or random-effects summary effects are likely to be biased and overestimate the true effect. Even the effect size from the largest trial in the meta-analysis may be biased sometimes, and often there may be no large enough trial. Consequently, the plausible effects used are conservative in testing for excess statistical significance. Moreover, because a random-effects meta-analysis gives larger relative weights to smaller trials than does a fixed-effect meta-analysis, the random-effects summary may be the farthest from the true effect in the presence of reporting bias. This may explain why the power of the proposed test was poorer when using the random-effects summaries as plausible effects. Finally, we explored the use of the single most precise trials and found that the proposed test had fair power. Therefore, for applications at the network level, we propose using either the result of the largest (most precise) trial or the fixed-effect summary. The former choice may often have a minor advantage.

The proposed test relied on further assumptions. We assumed that the observed number of trials with significant findings could be modelled by a common binomial parameter. However, because trial size varies within each meta-analysis, because the numbers of trials and the numbers of trials with significant findings vary across the meta-analyses constituting the network, the distribution of the total number of trials with significant findings is more complex. Moreover, we estimated the plausible effects based only on direct evidence and by considering that different comparisons in the network were independent. Other options may be considered for plausible effects. First, estimates from a consistency network meta-analysis model may be used as plausible effects. In the examples of antidepressant and antipsychotic trials, there were no closed loops, so this would not be relevant. But in cases where there are closed loops and network meta-analysis estimates can be obtained, it would be useful to compare the results of the excess significance test using also the network meta-analysis estimates as plausible effects in a sensitivity analysis. However, differential reporting bias may lead to violation of the consistency assumption [23]. The biases may operate in different directions across the meta-analyses constituting the network and biases could theoretically be cancelled out or exacerbated when indirect comparisons are made. Moreover, a single meaningful network meta-analysis is not feasible for many networks of trials (without any closed loop or in cases of disconnected sets of trial comparisons). Second, our simulation studies based on the Copas model showed that the arcsine transformation could outperform other options in a conventional meta-analysis [9]. But this metric, which concern dichotomous outcomes only, is not widely used in the literature yet and it cannot be used to combine results in network meta-analysis. However, by extension it is possible to apply the arcsine transformation to all the pairwise meta-analyses of a network for the purpose of excess significance testing. This approach may improve the method to maintain type 1 error rates.

A potential concern with the proposed test is that reporting bias and between-trial heterogeneity may be confounded. In fact, the type I error rate was inflated with increased between-trial variance. This is a typical issue with all tests of reporting bias introduced for conventional meta-analysis. However, we observed this finding with between-trial variance equal to 0.25 (only 25% of meta-analyses have this extent of heterogeneity [34]) and when assuming that all meta-analyses constituting the network had this amount of between-trial heterogeneity, which may be infrequent in practice. In such cases, all the evidence should perhaps be reconsidered before synthesis; that is, sources of heterogeneity should be explored and the test may still be applied considering a subset of more homogeneous trials. As well, it should be noted that reporting bias practices per se may induce between-trial heterogeneity [45, 46]. Regardless, the test should be used and interpreted wisely. In cases of a statistically significant signal of bias, one should not conclude that this bias is specifically publication bias, since it could reflect other practices such as selective analysis and outcome reporting biases. In the absence of significant signals of bias, one should not exclude the possibility of reporting bias, because a ŞnegativeŤ test result typically does not greatly decrease the prior odds of bias, unless in cases of many trials or substantial between-trial heterogeneity [47].

Although we illustrated the test with star-shaped networks of trials, the test can be used for networks of trials with closed loops. In this regard, a particular strength of our simulation studies is that the sets of realizations could reflect networks of placebo-controlled trials, head-to-head trials or both placebo-controlled and head-to-head trials. Moreover, multi-arm trials are frequent in networks of trials. The proposed test may handle them if a *K*-arm trial is analysed as *K*(*K*-1)/2 independent two-arm trials, with variances adjusted to compensate for the correlation between the corresponding estimates [48].

Our simulation studies have several limitations. First, values of variables were derived from a large sample of Cochrane meta-analyses to have realistic scenarios [33, 34]. However, we cannot exclude that some meta-analyses in this sample were affected by reporting biases. Reporting bias may have influenced the observed numbers of trials, trial sample sizes and between-trial heterogeneity [46]. Second, we found that the type I error and power of the proposed test varied depending on characteristics of the network known to the meta-analysts and other characteristics which will be always unknown, such as the nature of the trial selection mechanism. We addressed this issue by generating data under two previously used selection models: by trial size and intensity of treatment effect [9, 31, 32] or by p-value for the treatment effect associated with the trial [5, 7] and we found similar results. Of note, the proposed test does not directly make assumptions about small-study effects, whereas the Copas model assumes that trial selection depends upon trial size. We used this approach to estimate the operating characteristics of the test through a data generation process based on a plausible and realistic model for reporting bias but that did not fit exactly the principle underlying the test, i.e., significance chasing. Third, we considered true event rates between 0.3 and 0.7. The test may lose power when event rates are lower (or, equivalently, very high), but no test can have very good performance under such conditions. If a few meta-analyses in a network have event rates in the 0.3-0.7 range, these would be the ones primarily contributing to the power of the test. Fourth, we used a normal approximation to the likelihood, instead of the exact binomial likelihood, in the modeling for meta-analysis. This approximation is less precise for probabilities of events close to 0 or 1. Lastly, we did not consider the network geometry for our simulation. Instead, the simulation relied on some hyperdistribution of the true effect sizes for all comparisons.

Some other modeling approaches have been introduced recently to investigate the extent of reporting bias in a network meta-analysis. Mavridis et al. presented a Bayesian implementation of the Copas selection model extended to network meta-analyses [49]. The correlation parameter *ρ* is assumed equal for all comparisons and indicates the extent of reporting bias. Moreover, network meta-regression models that allows the treatment effect size to depend on its standard error or variance have been described [44, 50]. Under an assumption of exchangeable bias, a posterior mean slope is estimated and indicates the extent of small-study effect across the network. Other types of biases in a network, such as novelty bias, can be modeled with meta-regressions [51]. Some biases may affect not only a single network, but may be exchangeable across multiple networks (eg, all networks of systemic treatments for diverse types of cancer may suffer from similar biases, since more or less the same drugs and regimens are used for different cancers [51]). In these cases, one could apply the excess significance test across a collection of several such networks with exchangeable biases.

## Conclusion

The proposed excess significance test could be useful to provide a statistical signal indicating an excess of significant findings in clinical trial networks. If such a signal is detected across the network of trials or in specific pairwise comparisons by conventional approaches, the network of trials and its meta-analyses should be considered with caution.

## Electronic supplementary material

Additional file 1: **Protocol of simulation studies.** (DOCX 38 KB)

Additional file 2: **Results of simulation studies for conventional meta-analysis.** (DOCX 443 KB)

Additional file 3: **Additional results of simulation studies for trial networks.** (DOCX 89 KB)
