# Supplementary material for: A test for reporting bias in trial networks: simulation and case studies
Source: BMC Med Res Methodol. 2014 Sep 27;14:112. doi: 10.1186/1471-2288-14-112 (PMC4193287; doi:10.1186/1471-2288-14-112)
Supplement: Supplementary file 1 — Additional file 1: Protocol of simulation studies. (DOCX 38 KB) [file 12874_2014_1126_MOESM1_ESM.docx]

Protocol of simulation studies

We assessed the type I and type II error rates of the tests using Monte Carlo simulation studies. These simulations were based on binary outcome data. First, we assessed the performance of the test in conventional meta-analysis. Second, we assessed the performance of the generalized test in network meta-analysis.

1 Methods for generating the datasets

Simulation of trials in a meta-analysis

For a given meta-analysis (i.e., a given pair of experimental and control treatments), we generated the number of events and non-events in the experimental and control groups for each trial under a random-effects model.

We set the true average treatment effect as *θ* and the between-trial variance *τ*². Let us assume that *θ* <1 indicates benefit from the experimental treatment relative to the control treatment.

We drew the true underlying probabilities of events in the experimental and control groups for trial *i* as: logit(*p*_E_*_i_*)=logit(*p_i_*)+log(*θ*)+*τ*·*η_i_*/2 and logit(*p*_C_*_i_*)=logit(*p_i_*)-*τ*·*η_i_*/2, where logit(*x*)=log[*x*/(1-*x*)], *p_i_* was drawn from an uniform distribution on the interval [0.3; 0.7] and *η_i_* was drawn from a standard normal distribution. This distribution for the ‘true’ control group risk *p_i_* could be modified to reflect lower or higher risks.

In a sample of 77,237 trials included in 14,886 Cochrane meta-analyses with binary outcome data, the median sample size was 102 and the 75th percentile was 243 subjects [[1](#_ENREF_1)]. Consequently, we drew the log sample size for trial *i* from a normal distribution with mean 4.62 and standard deviation 1.29. Sample sizes < 20 were considered equal to 20. The sample size for trial *i* was further rounded to the nearest even number as *n_i_* = floor(*n_i_*)+ floor(*n_i_*) mod 2, with floor(*x*) the largest integer not greater than *x* and mod the modulo operation. Sample size was further assumed equal in the experimental and control groups, *n*_E_*_i_*=*n*_C_*_i_*=*n_i_*/2. The observed numbers of events *x*_E_*_i_* and *x*_C_*_i_* in the experimental and control groups of trial *i* were drawn from binomial distributions of parameters (*n*_E_*_i_*, *p*_E_*_i_*) and (*n*_C_*_i_*, *p*_C_*_i_*), respectively. If either no or all events occurred in both groups, the trial was excluded and replaced by a newly generated trial. If either no or all events occurred in one group only, 0.5 was added to all cells of the 2 ×2 table for the corresponding trial. These options represent standard practice to deal with trials with no events or with zero-cell counts [[2](#_ENREF_2)].

With this generation procedure, a random-effects meta-analysis model would be correctly specified as follows

log(*OR_i_*)=log(*θ_i_*)+*s_i_*·*ε_i_*, *ε_i_*~N(0,1) and log(*θ_i_*)~N(log(*θ*), *τ²*) (1)

because the log odds ratio for trial *i* can be estimated as log(*OR_i_*) = log[(*x*_E_*_i_*/*n*_E_*_i_*)/(*x*_C_*_i_*/*n*_C_*_i_*)] and its variance as *s_i_*²=1/*x*_E_*_i_*+1/(*n*_E_*_i_-x*_E_*_i_*)+1/*x*_C_*_i_*+1/(*n*_C_*_i_*-*x*_C_*_i_*).

Simulation of reporting bias in a meta-analysis

We used 2 selection models to simulate reporting bias affecting trials in a meta-analysis. The first one links the probability of trial selection to both trial size and intensity of treatment effect [[3-5](#_ENREF_3)]. The second one selects trials depending on the p-value for treatment effect associated with the trial [[6-8](#_ENREF_6)].

Model of selection of trials by trial size and treatment effect magnitude

We considered that model (1) would describe all existing trials but only some of them are selected in the meta-analysis with propensity

*z_i_*=*a*+*b*/*s_i_*+*δ_i_* with *δ_i_*~N(0,1) (2)

Only trials with positive values of *z_i_* are selected in the meta-analysis. Models (1) and (2) are combined as

log(*OR_i_*)=log(*θ_i_*)+*σ_i_*·*ε_i_*, *ε_i_*~N(0,1) with log(*θ_i_*)~N(log(*θ*), *τ²*), *z_i_*=a+b/*s_i_*+*δ_i_*, *δ_i_*~N(0,1)

and by assuming that the residuals (*ε_i_*, *δ_i_*) follow a bivariate normal distribution with both means 0, both variances 1 and correlation *ρ*. The standard deviation *σ_i_* of log(*OR_i_*) now differs from *s_i_* which is an estimate of the conditional variance of log(*OR_i_*) given that trial *i* was selected; *σ_i_* was estimated by $\frac{\text{2}}{\text{n}_{\text{i}}}\left( \frac{\text{1}}{\text{p}_{\text{E}\text{i}}}\text{+}\frac{\text{1}}{\text{1-}\text{p}_{\text{E}\text{i}}}\text{+}\frac{\text{1}}{\text{p}_{\text{C}\text{i}}}\text{+}\frac{\text{1}}{\text{1-}\text{p}_{\text{C}\text{i}}} \right)$. If *ρ* = 0, the selection is independent from the observed outcome; because we assumed that log(*θ*)<0 indicates benefit from the experimental treatment, when *ρ* is negative and large, selected trials have *z_i_* > 0 and are more likely to have positive *δ_i_*, thus negative *ε_i_* and log(*OR_i_*) underestimating log(*θ*), thereby showing larger benefit.

Under this model, the probability for trial *i* to be selected is shown as $\tilde{p}_{i}=\Phi\left( \left[ u_{i}+\tilde{\rho}_{i}\left( \left( \log\left( {OR}_{i} \right)-log(\theta) \right)/{\sqrt{\tau^{2}+\sigma_{i}^{2}}} \right) \right]/{\sqrt{1-\tilde{\rho}_{i}^{2}}} \right)$ where *u_i_*= *a*+*b*/*s_i_*, and $\tilde{\rho}_{i}={\sigma_{i}\cdot\rho}/{\sqrt{\tau^{2}+\sigma_{i}^{2}}}$.

We set (*a*, *b*) by setting arbitrary values for the selection probabilities for a smaller trial and a larger trial: P(trial *i* selected | *s_i_*=0.4)=0.1 and P(trial *i* selected | *s_i_*=0.05)=0.9, which gives *a*=-1.65 and *b*=0.16. Several values for *ρ* were considered (−0.6,−0.8,−1.0). Given log(*OR_i_*) and *s_i_*, it allows for estimating $\tilde{p}_{i}$. We drew a Bernoulli variable distributed with probability $\tilde{p}_{i}$. If the generated value is 1, the trial is selected; otherwise the trial is excluded and replaced with a newly generated trial.

*Model of selection of trials by treatment effect p-value*

We also used another class of explicit selection models proposed so far for meta-analysis to model reporting bias [[9-11](#_ENREF_9)]. The standard formulation of this selection model is that the probability density function of the observed treatment effect $y_{i}=log\left( {OR}_{i} \right)$ is a weighted density of the treatment effect before selection: $f\left( y_{i} | \mathrm{trial}i selected,s_{i} \right)={f\left( y_{i} | s_{i} \right)\cdot P\left( \mathrm{trial}i\mathrm{selected} | y_{i},s_{i} \right)}/{\int_{-\infty}^{\infty} f\left( y_{i} | s_{i} \right)\cdot P\left( \mathrm{trial}i\mathrm{selected} | y_{i},s_{i} \right)dy_{i}}$, where $f\left( y_{i} | s_{i} \right)$ is the density function of the treatment effect before selection (a normal distribution). If the weight function $P\left( \mathrm{trial}i\mathrm{selected} | y_{i},s_{i} \right)$ is not a constant, the distribution of the observed treatment effect differs from that of the unselected treatment effect, and this difference corresponds to reporting bias. More specifically, we considered that only some trials are selected in the meta-analysis according to a known weight function $P\left( \mathrm{trial}i\mathrm{selected} | y_{i},s_{i} \right)=w\left( p_{i} \right)=exp(-\gamma_{0}p_{i}^{\gamma_{1}})$ with *p_i_* the p-value associated with a two-sided Fisher’s exact test for a difference in the proportion of events between the experimental and control groups of trial *i*. Because *w*(*p_i_*) is a non-increasing function of the p-value, trials with smaller p-values are more likely to be selected than are those with larger p-values. Several values for ($\gamma_{0}$, $\gamma_{1}$) were considered ($\gamma_{0}$=4 and $\gamma_{1}$= 3, 3/2 or 3/4). Because we set $\gamma_{0}$ and $\gamma_{1}$, it allowed for estimating *w*(*p_i_*). For each trial, we drew a Bernoulli variable distributed with probability *w*(*p_i_*). If the generated value was 1, the trial is selected, otherwise the trial is excluded and replaced with a newly generated trial.

Simulation of a network of trials

We simulated a network of trials as *J* meta-analyses of *n_j_*=*n* trials each. For each meta-analysis *j*, we set the true average treatment effect *θ_j_*. We considered that the *J* true average treatment effects would be centered on a log odds ratio of *ψ_1_* =log(0.75) or *ψ_2_* =log(0.95). We further considered that the dispersion of the true average treatment effects across the *J* meta-analyses could be smaller or larger, with variance on the log scale of *ν_1_*=0.02 and *ν_2_*=0.08, respectively. In all, 95% of true average odds ratios across the *J* meta-analyses would be within a factor of exp(1.96×√*ν*) of *ψ*. This equates to factors of 1.32 and 1.74 when the variance is *ν_1_* and *ν_2_*, respectively. Consequently, we considered 4 distinct scenarios by setting known relative effects from log(*θ_j_*)~N(*ψ*, *ν*) with *ψ* = *ψ_1_* or *ψ_2_* and *ν* = *ν_1_* or *ν_2_*. The sets of realizations are reported in Table 1. We further assumed homogeneity of between-trial variation within the *J* meta-analyses, i.e., *τ_j_*²= *τ*². For a given meta-analysis, data were then generated as described.

Simulation of reporting bias in a network of trials

Reporting bias was induced for each of the *J* meta-analyses constituting the network as described, under the assumption that the propensity for bias would be similar across the *J* meta-analyses. When selecting trials according to trial size and treatment effect magnitude, we drew each *ρ_j_* from an uniform distribution over the support [*ρ_min_*;*ρ_max_*]. When selecting trials according to treatment effect p-value, we drew each $\gamma_{1j}$ from a uniform distribution over the support [$\gamma_{1min}$;$\gamma_{1max}$]. Several values were considered for (*ρ_min_*, *ρ_max_*) and ($\gamma_{1min}, \gamma_{1max}$) and are thus given below in the section describing the various scenarios.

2 Scenarios to be investigated

In a sample of 77,237 trials included in 14,886 Cochrane meta-analyses with binary outcome data, the median number of trials in a meta-analysis was 3 (75% percentile 6, 90% percentile 10 and 99% percentile 28) [[1](#_ENREF_1)]. From the same sample of meta-analyses, the predictive distribution for between-trial heterogeneity in a future meta-analysis in a general setting was estimated as lognormal (-2.56,1.74) for median 0.08 and 25%-75% percentiles 0.02-0.25 on the untransformed scale [[12](#_ENREF_12)]. Consequently, we considered the following scenarios.

To simulate a meta-analysis, we chose *n* as the number of trials, the true average treatment effect *θ* and between-trial variance *τ*². We used *n* as (6, 10, 30); *θ* as (0.5, 0.75, 1.0); *τ*² as (0.02, 0.08, 0.25). When we induced reporting bias, we simulated data until *n* trials had been selected with *ρ* as (−0.6,−0.8,−1.0) or with $\gamma_{0}$=4 and $\gamma_{1}$as (3, 3/2, 3/4). These values correspond to different extents of bias from moderate to severe.

To simulate a network of trials, we chose *J* as the number of meta-analyses constituting the network, *n_j_*=*n* the number of trials in each meta-analysis, the true average treatment effects *θ_j_* and the between-trial variation *τ*². We took *J* as (6, 10), *n* as (3,6), *θ_j_* as in Table 1, and *τ*² as (0.02, 0.08, 0.25). When we induced reporting bias, we simulated data until *n* trials had been selected with each *ρ_j_*~Uniform[−0.8;−0.6] or *ρ_j_*~Uniform[−1.0;−0.8] or with $\gamma_{0j}$=4 and $\gamma_{1j}$~Uniform[3/2;3] or $\gamma_{1j}$~Uniform[3/4;3/2] to reflect similar moderate and severe bias, respectively, across the *J* meta-analyses.

3 Criteria to evaluate the performance of the test

We assessed the performance of the tests in a conventional meta-analysis and in a network meta-analysis. We assessed its empirical type I error rate and power (1- type II error rate) for scenarios without and with reporting bias, respectively. Each test statistic was simulated *B* times under the null hypothesis and *B* times under the alternative. For a network meta-analysis, the estimated type I error rate is $\hat{\alpha}=B^{-1}\#\left\{ \sum_{i=O}^{N} b(\left. i \right|N,E/N)<0.10 | no reporting bias \right\}$ and the estimated power is $1-\hat{\beta}=B^{-1}\#\left\{ \sum_{i=O}^{N} b(\left. i \right|N,E/N)<0.10 | reporting bias \right\}$.

Because the estimated type I error rate can differ from the nominal type I error level, we took into account the possibly differing type I error rates of the tests and we estimated powers adjusted for type I error rate by a probit analysis [[13](#_ENREF_13)]. Moreover, we estimated the likelihood ratio of a positive test result as the ratio of the (unadjusted) power to the type I error rate $\left( 1-\hat{\beta} \right)/\hat{\alpha}$. This reflects the likelihood that a significant test result is found in networks with bias as opposed to networks without bias. We also estimated the likelihood ratio of a negative test result as the ratio of the (unadjusted) type II error rate to 1 minus the type I error rate $\hat{\beta}/\left( 1-\hat{\alpha} \right)$. This reflects the likelihood that a non-significant test result is found in networks with bias as opposed to networks without bias.

In the cases of a conventional meta-analysis, we also assessed for comparison purposes the performance of the test introduced by Rucker et al*.* based on a weighted regression of the arcsine transformation of observed risks with explicit modeling of between-trial heterogeneity [[5](#_ENREF_5)].

4 Number of simulations to be performed

To obtain a confidence interval, with confidence level 1-*α*, for proportion *p* with pre-specified accuracy *A*, the required number of simulations *B* is *z*²_1-_ *_α_*_/2_·*p*·(1 - *p*)/*A*² with *z*_ξ_ the ξth percentile of the standard normal distribution. For a pre-specified accuracy A, the latter will reach its maximum for *p*=0.5. For each scenario, we consequently generated *B*=10,000 datasets, which would guarantee a precision of ≤1% on the estimation of the power of the test with confidence 95%. Moreover, with such a number of simulations, an empirical type I error rate ≤9.4% or ≥ 10.6% would be considered a departure from the pres-specified 10% nominal level.

Analyses involved use of R v2.12.2 (R Development Core Team, Vienna, Austria).

**References**

1. Davey J, Turner RM, Clarke MJ, Higgins JP: **Characteristics of meta-analyses and their component studies in the Cochrane Database of Systematic Reviews: a cross-sectional, descriptive analysis**. *BMC Med Res Methodol* 2011, **11**:160.

2. Higgins JP, Deeks JJ, Altman DG: **Chapter 16: Special topics in statistics**. In *Cochrane Handbook for Systematic Reviews of Interventions*. Edited by Higgins JP, Green S: The Cochrane Collaboration; 2011.

3. Copas J, Shi JQ: **Meta-analysis, funnel plots and sensitivity analysis**. *Biostatistics* 2000, **1**(3):247-262.

4. Copas JB, Shi JQ: **A sensitivity analysis for publication bias in systematic reviews**. *Stat Methods Med Res* 2001, **10**(4):251-265.

5. Rucker G, Schwarzer G, Carpenter J: **Arcsine test for publication bias in meta-analyses with binary outcomes**. *Stat Med* 2008, **27**(5):746-763.

6. Begg CB, Mazumdar M: **Operating characteristics of a rank correlation test for publication bias**. *Biometrics* 1994, **50**(4):1088-1101.

7. Harbord RM, Egger M, Sterne JA: **A modified test for small-study effects in meta-analyses of controlled trials with binary endpoints**. *Stat Med* 2006, **25**(20):3443-3457.

8. Macaskill P, Walter SD, Irwig L: **A comparison of methods to detect publication bias in meta-analysis**. *Stat Med* 2001, **20**(4):641-654.

9. Hedges LV, Vevea J: **Selection method approaches**. In *Publication bias in meta-analysis*. Edited by Rothstein H, Sutton AJ, Borenstein M. Chichester: John Wiley & Sons Ltd; 2005.

10. Dear KB, Begg CB: **An Approach for Assessing Publication Bias Prior to Performing a Meta-Analysis**. *Statist Sci* 1992, **7**(2):237-245.

11. Iyengar S, Greenhouse JB: **Selection Models and the File Drawer Problem**. *Statist Sci* 1988, **3**(1):109-117.

12. Turner RM, Davey J, Clarke MJ, Thompson SG, Higgins JP: **Predicting the extent of heterogeneity in meta-analysis, using empirical data from the Cochrane Database of Systematic Reviews**. *Int J Epidemiol* 2012, **41**(3):818-827.

13. Lloyd CJ: **Estimating test power adjusted for size**. *J Stat Comput Simulat* 2005, **75**(11):921-933.
