# Supplementary material for: A test for reporting bias in trial networks: simulation and case studies
Source: BMC Med Res Methodol. 2014 Sep 27;14:112. doi: 10.1186/1471-2288-14-112 (PMC4193287; doi:10.1186/1471-2288-14-112)
Supplement: Supplementary file 2 — Additional file 2: Results of simulation studies for conventional meta-analysis. (DOCX 443 KB) [file 12874_2014_1126_MOESM2_ESM.docx]

**Results of simulation studies for conventional meta-analysis**

1 Type I error rate

Results for scenarios without reporting bias are presented in Figure S1. Visual inspection of the trellis graph showed that the type I error rate increased with increasing heterogeneity and increasing number of trials per meta-analysis. When the true effect size was estimated as the fixed-effect or random-effects summary, the proposed test was too conservative in cases with 6 or 10 trials, a true odds ratio of 0.5 or 0.75 and between-trial variance of 0.02 or 0.08. However, the proposed test showed substantial error inflation when heterogeneity was substantial (between-trial variance of 0.25) and/or with a large number of trials per meta-analysis (30 trials), with estimated type I error rate ≥ 50%. When the true effect size was estimated as the treatment effect estimate of the largest trial in the meta-analysis, the empirical type I error rate was in agreement with the pre-specified significance level of 0.10, except when the number of trials was large (30 trials), whatever the heterogeneity, or in cases of 10 trials with between-trial variance 0.02 or 0.08, with the estimated type I error rate ranging from 14.9% to 39.4%. Finally, the arcsine test introduced by Rucker et al*.* maintained a better type I error rate, ranging from 3.4% to 12.9% across all scenarios. The test was too conservative in cases of low heterogeneity and number of trials, otherwise the empirical type I error rate was in good agreement with the pre-specified significance level of 0.10.

2 Power

Results for scenarios with reporting bias modeled according to trial size and treatment effect magnitude (with *ρ*=−0.8) are presented in Figure S2. Visual inspection of the trellis graphs showed that the power adjusted for type I error rate increased with increasing number of trials per meta-analysis and with decreasing heterogeneity. With low number of trials per meta-analysis, all tests had low power. When selection of trials was modeled by trial size and treatment effect magnitude, the power increased when the odds ratio moved away from 1. The most powerful test was the arcsine test. When the true odds ratio was 0.5, the heterogeneity low or moderate and the number of trials large, the proposed test, with the true effect size estimated as the fixed-effect or random-effects summary, had lower but acceptable power. In all other cases, because of the inflation of the type I error rate, the power adjusted for type I error rate of the proposed test was clearly insufficient. Results for other extents of bias were similar (Appendix, Figure S4 and S5). When selection of trials was modeled by p-value, the most powerful test was the arcsine test only when the true odds ratio was 0.5. The proposed test, with the true effect size estimated as the fixed-effect or random-effects summary, had acceptable power when the true odds ratio was 1 (Appendix, Figure S6-S8).

3 Likelihood ratio

Likelihood ratios of a positive test result indicated that the proposed test had a moderate effect on increasing the likelihood of bias (Figure S3). Likelihood ratios of a negative test result indicated that the proposed test had a weak effect on decreasing the likelihood of bias. The arcsine test yielded the best likelihood ratios. The proposed test with the true effect size estimated as the random-effects summary had the worst ability to change the likelihood of bias.

Figure S1:type I error rate of the tests for reporting bias in a conventional meta-analysis


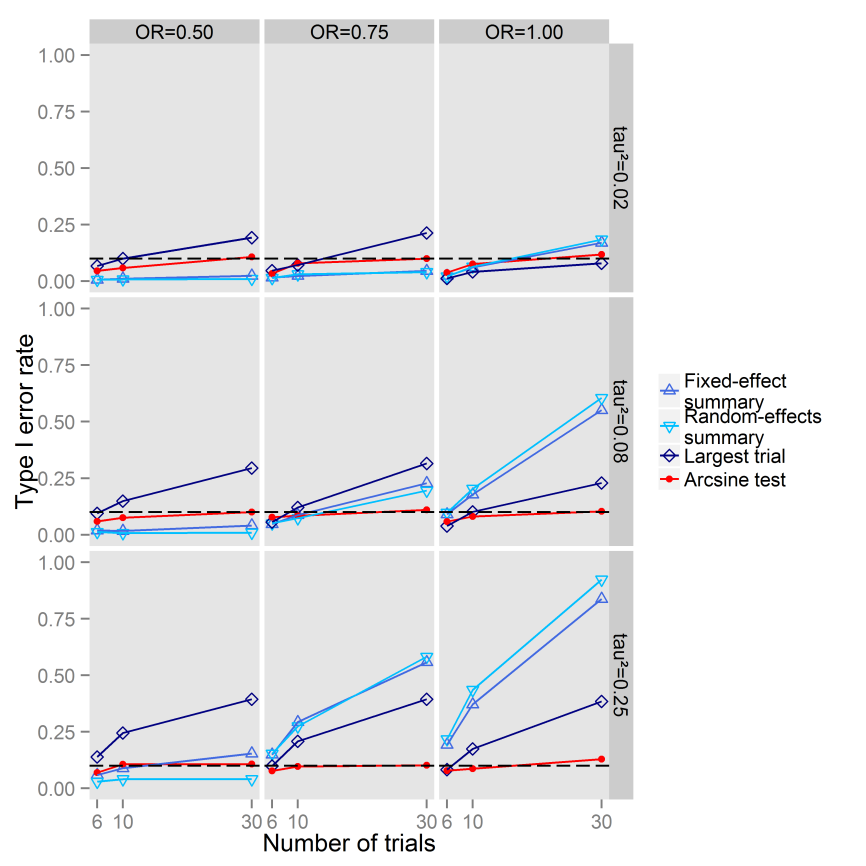


Figure S2: adjusted power of the tests for reporting bias in a conventional meta-analysis (trial selection modeled by trial size and intensity of treatment effect with *ρ*=−0.8)


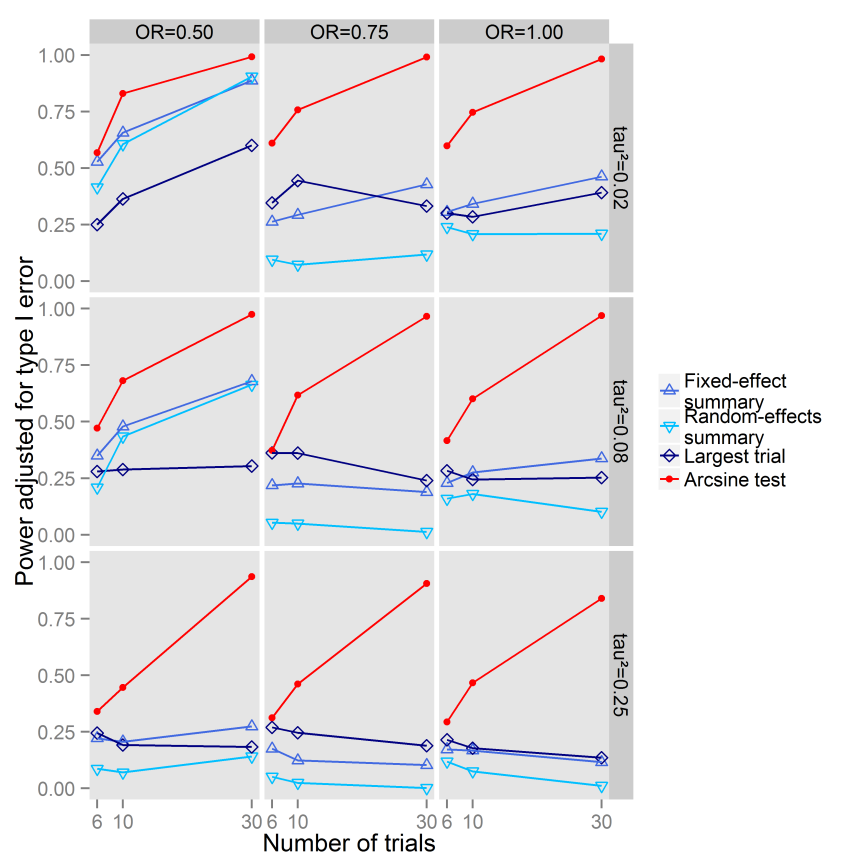


Figure S3: Adjusted power of the tests for reporting bias in a conventional meta-analysis (trial selection modeled by trial size and intensity of treatment effect with ρ=−0.6)

Figure S4: Adjusted power of the tests for reporting bias in a conventional meta-analysis (trial selection modeled by trial size and intensity of treatment effect with ρ=−1)

Figure S5: Adjusted power of the tests for reporting bias in a conventional meta-analysis (trial selection modeled by p-value associated with the treatment effect with γ_0=4 and γ_1=3)

Figure S6: Adjusted power of the tests for reporting bias in a conventional meta-analysis (trial selection modeled by p-value associated with the treatment effect with γ_0=4 and γ_1=3/2)

Figure S7: Adjusted power of the tests for reporting bias in a conventional meta-analysis (trial selection modeled by p-value associated with the treatment effect with γ_0=4 and γ_1=3/4)
