# Supplementary material for: A test for reporting bias in trial networks: simulation and case studies
Source: BMC Med Res Methodol. 2014 Sep 27;14:112. doi: 10.1186/1471-2288-14-112 (PMC4193287; doi:10.1186/1471-2288-14-112)
Supplement: Supplementary file 3 — Additional file 3: Additional results of simulation studies for trial networks. (DOCX 89 KB) [file 12874_2014_1126_MOESM3_ESM.docx]

**Additional results of simulation studies for a network of trials**

Figure S8: Adjusted power of the extended tests for reporting bias in a network meta-analysis (trial selection modeled by trial size and intensity of treatment with ρj~Uniform[−0.8;−0.6])

Figure S9: Adjusted power of the extended tests for reporting bias in a network meta-analysis (trial selection modeled by p-value associated with treatment effect with γ_0j=4 and each γ_1j~Uniform[3/2;3])

Figure S10: Adjusted power of the extended tests for reporting bias in a network meta-analysis (trial selection modeled by p-value associated with treatment effect with γ_0j=4 and each γ_1j~Uniform[3/4;3/2])
